# AT2R activation increases in vitro angiogenesis in pregnant human uterine artery endothelial cells

**DOI:** 10.1371/journal.pone.0267826

**Published:** 2022-04-29

**Authors:** Jay S. Mishra, Dong-Bao Chen, Sathish Kumar

**Affiliations:** 1 Department of Comparative Biosciences, School of Veterinary Medicine, University of Wisconsin, Madison, Wisconsin, United States of America; 2 Department of Obstetrics and Gynecology, University of California, Irvine, California, United States of America; 3 Department of Obstetrics and Gynecology, School of Medicine and Public Health, University of Wisconsin, Madison, Wisconsin, United States of America; 4 Endocrinology-Reproductive Physiology Program, University of Wisconsin, Madison, Wisconsin, United States of America; Normandie Universite, UNITED STATES

## Abstract

Angiogenesis is vital during pregnancy for remodeling and enhancing vasodilation of maternal uterine arteries, and increasing uterine blood flow. Abnormal angiogenesis is associated with decreased uteroplacental blood flow and development of pregnancy disorders such as gestational hypertension, preeclampsia, fetal growth restriction, preterm delivery, stillbirth, and miscarriage. The mechanisms that contribute to normal angiogenesis remain obscure. Our previous studies demonstrated that expression of the angiotensin type 2 receptor (AT2R) is increased while the angiotensin type 1 receptor (AT1R) is unchanged in the endothelium of uterine arteries, and that AT2R-mediated pregnancy adaptation facilitates enhanced vasodilation and uterine arterial blood flow. However, the role of AT2R in regulating angiogenesis during pregnancy has never been studied. This study examines whether or not AT2R activation induces angiogenesis and, if so, what mechanisms are involved. To this end, we used primary human uterine artery endothelial cells (hUAECs) isolated from pregnant and nonpregnant women undergoing hysterectomy. The present study shows that Compound 21, a selective AT2R agonist, induced proliferation of pregnant-hUAECs, but not nonpregnant-hUAECs, in a concentration-dependent manner, and that this C21-induced mitogenic effect was blocked by PD123319, a selective AT2R antagonist. The mitogenic effects induced by C21 were inhibited by blocking JNK—but not ERK, PI3K, and p38—signaling pathways. In addition, C21 concentration dependently increased cell migration and capillary-like tube formation in pregnant-hUAECs. The membrane-based antibody array showed that C21 increased expression of multiple angiogenic proteins, including EGF, bFGF, leptin, PLGF, IGF-1, and angiopoietins. Our qPCR analysis demonstrates that C21-induced increase in expression of these angiogenic proteins correlates with a proportional increase in mRNA expression, indicating that AT2R activates angiogenic proteins at the transcriptional level. In summary, the present study shows that AT2R activation induces angiogenesis of hUAECs in a pregnancy-specific manner through JNK-mediated pathways with associated transcriptional upregulation of multiple proangiogenic proteins.

## Introduction

Angiogenesis (growth of new blood vessels from pre-existing vasculature) and vascular remodelling (including branching, enlargement and network formation) are process is crucial during pregnancy to deliver sufficient oxygen and nutrients to the uterus as well as to the embryo [1]. Specifically, the endothelial cells in the uterine artery undergo proliferation, differentiation, and migration to establish a “low resistance, high capacitance vessel” capable of increasing the uterine blood flow by 53 fold compared to the nonpregnant state [2, 3]. Abnormal angiogenesis is asscoaited with decreased uterine blood flow and development of pregnancy disorders such as gestational hypertension, preeclampsia, fetal growth restriction, preterm delivery, stillbirth, and miscarriage [4, 5]. Therefore, proper establishment and remodeling of blood vessels within the uterine artery is central to fetal growth and survival, and understanding the angiogenic mechanisms may help develop therapeutic strategies for mitigating clinical conditions associated with altered angiogenesis [4].

Angiogenic stimuli during pregnancy are generated through activation of endothelial cell signaling and gene transcription of key molecules, such as vascular endothelial growth factor (VEGF) and placental growth factor (PIGF) [6]. VEGF promotes angiogenesis through interaction with 2 high-affinity receptors, VEGF receptor-1 (VEGFR-1, also known as Flt-1) and VEGF receptor-2 (VEGFR-2, also known as KDR/Flk-1). The production of antiangiogenic factors increases toward the end of gestation when continued expansion in increased blood flow is no longer needed. One such antiangiogenic factor is a soluble form of VEGFR-1 (sFlt-1) that binds to circulating VEGF and placental growth factors, inhibiting their angiogenic activities. More recently, angiogenesis and vascular remodeling was shown to be induced by angiotensin II (Ang II), a key regulator of blood pressure and the main effector of the renin–angiotensin-aldosterone system [7–9].

Ang II mediates its effect mainly through two types of receptors: the Ang II type 1 receptor (AT1R) and the Ang II type 2 receptor (AT2R). During normal pregnancy, plasma levels of Ang II progressively increase with advancing gestation with a proportional increase in AT2R and unchanged AT1R in the uterine arteries of rodents, sheep, and women [10–12]. For example, in pregnant rats and women, both the expression of AT2R mRNA and protein levels are approximately 4- and 6-fold greater, respectively, during pregnancy than in nonpregnant conditions [10]. This pregnancy-related increase in AT2R expression occurs specifically in endothelial cells of uterine arteries but not in other vascular beds [13]. Coincident with the increased AT2R expression, uterine blood flow increase dramatically during normal pregnancy [14]. Conversely, AT2R expression in placental vessels is decreased during preeclampsia and feto-placental growth restriction [10]. Knockout or blockade of AT2R in pregnant mice and rats induces preeclampsia-like features along with feto-placental growth restriction [14–16]. On the other hand, activation of AT2R with a selective agonist compound 21 (C21) in animal models of preeclampsia increase vasodilation, restore blood pressure, enhance uterine artery blood flow, and improve feto-placental growth [10]. C21 is a highly selective AT2R agonist with a K_i_ value of 0.4 nM for the AT1R and a K_i_ > 10 mM for the AT1R [17]. Although these studies indicate that the pregnancy-specific increase in AT2R expression is critical for uterine arterial remodeling and blood flow, the direct regulatory role of AT2R in angiogenesis in the uterine arteries is unknown. Herein, we hypothesize that AT2R positively induces angiogenesis. To test this hypothesis, we isolated human uterine artery endothelial cells (hUAECs) from normal nonpregnant and pregnant women, treated with a selective AT2R agonist C21, and examined measures of angiogenesis and underlying mechanisms. Our results show for the first time that AT2R activation promotes proliferation in pregnant-hUAECs but not nonpregnant-hUAECs through JNK pathways but not through ERK, p38, and PI3K pathways. Further, AT2R-induced angiogenesis in pregnant-hUAECs is associated with transcriptional upregulation of multiple proangiogenic proteins.

## Materials and methods

### hUAEC isolation and culture

We isolated hUAECs from the main uterine arteries of 30- to 45-year-old nonpregnant and pregnant (35–36 weeks of gestation; n = 5/group) women undergoing hysterectomy, as we had described previously [13, 18]. Written consent was obtained from all subjects, and ethical approval (HS#2013–9763) was granted by the Institutional Review Board at the University of California Irvine [18]. After collagenase digestion, endothelial cells were purified, validated, and cultured in endothelial cell medium (ECM; ScienCell, La Jolla, CA) containing 5% fetal bovine serum, endothelial growth supplements, and 1% penicillin/streptomycin and used within 4–5 passages. When cells reached 70% confluence, the medium was changed to M199 medium containing 0.1% BSA, 0.5% fetal bovine serum, 1% antibiotics, and 25 mmol/L HEPES and was used for experimental treatments.

### Proliferation assay

To assess the proliferation competency of hUAECs we used the MTS cell proliferation colorimetric assay kit (Biovision, Milpitas, CA) following the manufacturer’s instructions. Briefly, nonpregnant-hUAECs and pregnant-hUAECs (5 × 10^3^/well) were seeded in a 96-well plate treated with or without increasing concentrations of C21 (1, 10, and 100 nmol/L; C21 was kindly provided by Vicore Pharma, Gothenburg, Sweden) for 48 h. MTS reagent (20 μL) was added for the last 3 h, and we evaluated the *in vitro* index of proliferation by measuring optical density at 490 nm with a SpectraMax i3x multi-mode plate reader (Molecular Devices, San Jose, CA). We blocked AT2R by pretreating hUAECs for 30 min with 10 μmol/L of the AT2R antagonist PD123319 (Tocris, Minneapolis, MN). To determine the roles of extracellular signal‐regulated kinases (ERKs), p38 mitogen-activated protein kinase (p38 MAPK), c‐Jun N‐terminal kinase (JNK), and phosphatidylinositol 3‐kinase (PI3K) pathways, specific inhibitors were used. Cells were treated with C21 in the absence or presence of ERK1/2 inhibitor (PD98059, 10 μmol/L, Tocris), p38 MAPK inhibitor (SB303580, 10 μmol/L, Tocris), JNK inhibitor (SP600125, 10 μmol/L, Cayman, Ann Arbor, MI), and PI3K inhibitor (LY294002, 10 μmol/L, Cell Signaling Technology, Danvers, MA), and were processed for proliferation assay as described above.

### Cell-migration assay

We assessed endothelial cell migration by using an 8-μm pore-sized filter transwell (Corning FluoroBlok, Corning, NY) as described previously [19]. The inserts were coated with 20 μg/ml of fibronectin (Sigma-Aldrich, St. Louis, MO) and kept overnight at 4°C. The next day, hUAECs were plated on the upper chamber of the insert membrane, treated with increasing concentrations of C21 (1, 10, and 100 nmol/L) and incubated in 5% CO_2_ at 37°C. After 16 h, the number of cells that had migrated through the membrane to the lower chamber was measured with a fluorescent dye, calcein acetoxymethyl ester (Calcein-AM, 0.2 μg/ml; Invitrogen, Waltham, MA). The cells in the lower chamber were visualized with a BZ-X710 inverted microscope (Keyence, Osaka, Japan) and quantified with a bottom-reading SpectraMax i3x fluorescence plate reader (Molecular Devices).

### Tube-formation assay

“Growth factor”-reduced Matrigel (Corning FluoroBlok, Corning, NY) was added to a 48-well plate and incubated at 37°C for 30 min to allow for solidification. hUAECs were seeded (1 X 10^5^ cells per well) onto Matrigel-coated plates and incubated in the presence or absence of increasing concentrations of C21 (1, 10, and 100 nmol/L) and incubated for 8 h at 37°C. Images were obtained with a MicroPublisher 5.0 camera mounted on an Olympus SZX-10 stereomicroscope (Tokyo, Japan). Images of five randomly chosen fields of each well were captured. Then, with an ImageJ “Angiogenesis Analyzer” tool, we quantified tube formation by counting the total number of tube branches and averaged the values.

### Angiogenesis arrays

With antibody-based human angiogenesis array (AAH-ANG-1000; RayBiotech, Peachtree Corners, GA), we simultaneously detected 43 proteins, comprising growth and differentiation factors, membrane-bound receptors, extracellular matrix components, and intracellular signaling molecules (Table 1).

**Table 1:** Human angiogenesis antibody array proteins.

Angiogenin	PDGF-BB	Angiostatin	MCP-3
EGF	PlGF	Endostatin	MCP-4
ENA-78	RANTES	G-CSF	MMP-1
bFGF	TGF-beta 1	GM-CSF	MMP-9
GRO	TIMP-1	I-309	PECAM-1
IFN-gamma	TIMP-2	IL-10	Tie-2
IGF-I	Thrombopoietin	IL-1 alpha	TNF alpha
IL-6	VEGF	IL-1 beta	u PAR
IL-8	VEGF-D	IL-2	VEGF R2
Leptin	Angiopoietin-1	IL-4	VEGF R3
MCP-1	Angiopoietin-2	I-TAC	

The assay was performed as per the manufacturer’s instructions (RayBiotech) and as previously described [20]. Briefly, hUAECs were treated with C21 (10 nmol/L) for 24 h and then lysed with ice-cold radioimmunoprecipitation assay (RIPA) buffer (Cell Signaling Technology, Danvers, MA) containing protease and phosphatase inhibitors (Sigma). Lysates were cleared by centrifugation at 14,000 g at 4°C for 10 min, and protein concentration was determined with the BCA assay kit (Pierce; Thermo Scientific, Waltham, MA). The human angiogenesis array membranes were blocked with 2-ml blocking buffer for 30 min at room temperature. The membranes were incubated with 1 ml of 1:10 diluted cell lysates overnight at 4°C. The membranes were washed and incubated with primary antibody for 2 h at room temperature. After the membranes were washed again with a washing buffer, they were incubated with horseradish peroxidase-labeled anti-rabbit secondary antibody for 2 h. Membrane-bound proteins were detected with electrochemiluminescent detection reagents (Pierce; Thermo Scientific, Waltham, MA), and normalized band intensities were quantified with ImageJ and RayBiotech software tools.

### Quantitative real-time PCR

Cells were treated with C21 (10 nmol/L) for 24 h, and total RNA was isolated with an RNeasy mini kit (QIAGEN, Valencia, CA) as per the manufacturer’s instructions. The RNA integrity and concentrations were determined with a DS-11 spectrophotometer (DeNovix, Wilmington, DE), and cDNA synthesis was done with 1 μg of total RNA using an iScript cDNA synthesis kit (Bio-Rad, Hercules, CA). After dilution, we amplified cDNA corresponding to 20 ng of RNA by using qRT-PCR, a CFX96 real-time thermal cycler (Bio-Rad, Hercules, CA), and an SsoAdvanced Universal SYBR Green Supermix (Bio-Rad, Hercules, CA). Primer sequences are shown in Table 2. To analyze the data, we used the 2^-ΔΔCT^ method, and expressed the results as the fold change of the gene of interest in treated versus control samples. All reactions were performed in duplicate, and GAPDH was used as an internal control.

**Table 2:** Quantitative real-time PCR primer sequence.

Gene	Forward	Reverse
EGF	**GTGCAGCTTCAGGACCACAA**	**AAATGCATGTGTCGAATATCTTGAG**
ENA-78	**GCCCGTGTCCCCGGTCCTTCGAG**	**CTGGATCAAGACAAATTTCCTTC**
bFGF	**CAAGCGGCTGTACTGCAA**	**CCCAGGTCCTGTTTTGGA**
IGF-1	**CTTCAGTTCGTGTGTGGAGACAG**	**CGCCCTCCGACTGCTG**
Leptin	**TCCCCTCTTGACCCATCTC**	**GGGAACCTTGTTCTGGTCAT**
PLGF	**CAGAGGTGGAAGTGGTACCCTTCC**	**CGGATCTTTAGGAGCTGCATGGTGAC**
TIMP-1	**TCTGGCATCCTGTTGTTGCT**	**CGCTGGTATAAGGTGGTCTGG**
TIMP-2	**ATGCAGATGTAGTGATCAGG**	**CGTTGATGTTCTTCTCTGTG**
Angiopoetin-1	**GGGGGAGGTTGGACTGTAAT**	**AGGGCACATTTGCACATACA**
Angiopoetin-2	**TGGACAATTATTCAGCGACGTG**	**GCTGGTCGGATCATCATGGTTG**

### Statistical analysis

Data were analyzed with GraphPad Prism (GraphPad Software, San Diego, CA) presented as mean ± SD. Experiments were performed in triplicates and all experiments were repeated at least three to five times using cells from different subjects. Two groups were compared with unpaired Student t-tests. Multiple group comparisons were performed using ANOVA, followed by Newman–Keuls tests. Statistically significant differences were reported when p < 0.05.

## Results

### AT2R activation increased cell proliferation of pregnant-hUAECs, but not nonpregnant-hUAECs

**Fig 1:**
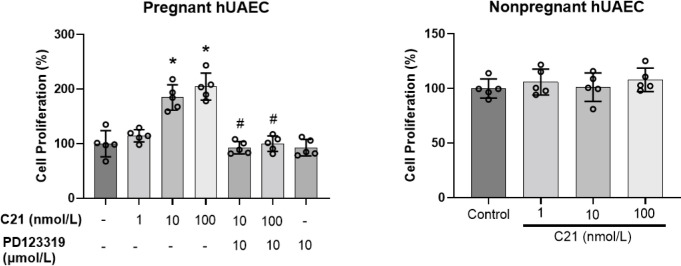
Effect of AT2R agonist C21 on proliferation of pregnant- and nonpregnant-hUAECs. Cells were treated with increasing concentrations of C21 (1–100 nmol/L) for 48 h with or without AT2R blocker PD123319 (10 μmol/L). Cell proliferation was measured with the MTS Cell Proliferation Assay kit. Data were expressed as mean ± SD from five independent experiments using hUAECs from five donors. *P < 0.05 vs control. ^#^P < 0.05 vs respective C21 concentration without PD123319.

In our effort to assess the effects of AT2R activation on cell proliferation, hUAECs isolated from pregnant and nonpregnant women were exposed to varying concentrations of C21 for 48 h. As shown in Fig 1, C21 induced proliferation of pregnant-hUAECs, but not nonpregnant-hUAECs, in a concentration-dependent manner. Increasing concentrations of C21 (1, 10, 100 nmol/L) resulted in increased cell proliferation by 14%, 84%, and 104%, respectively. We next assessed whether the effect of C21 was mediated via binding to its cognate receptor AT2R. As shown in Fig 1, AT2R selective antagonist-PD123319 blocked cell proliferation induced by C21 in pregnant-hUAECs. Overall, these findings indicate that C21 induces hUAEC proliferation through AT2R in a pregnancy-specific manner.

### JNK inhibitor blocked C21-stimulated cell proliferation

**Fig 2:**
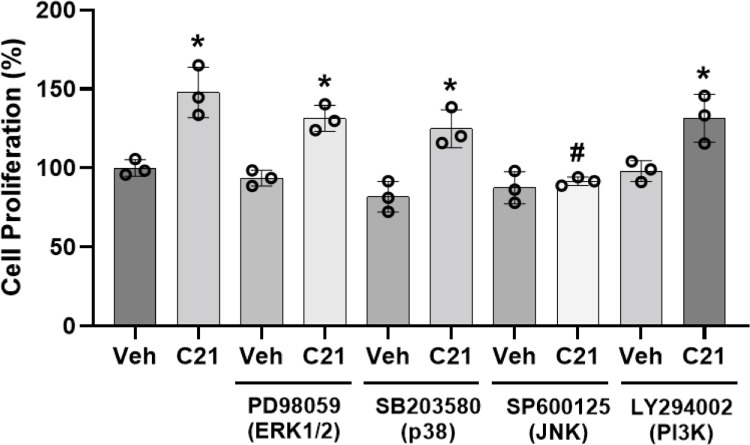
Effect of blockade of ERK1/2, p38, JNK, and PI3K on C21-induced proliferation of pregnant-hUAECs. Cells were pretreated with specific inhibitors for 30 min followed by treatment with C21 (10 nmol/L) for another 48 h. Cell proliferation was measured with an MTS Cell Proliferation Assay kit. Data were expressed as mean ± SD from three independent experiments using hUAECs from three donors and calculated as fold of control. *P < 0.05 vs control.

ERK, PI3K, p38, and JNK pathways have been demonstrated to play a crucial role in cell proliferation [21, 22]. Pretreatment with PD98059 (ERK1/2), SB303580 (p38), and LY294002 (PI3K) inhibitors did not have any effect, but JNK inhibitor SP600125 blocked C21-mediated proliferation of pregnant-hUAECs (Fig 2). Incubation with inhibitors alone did not affect cell proliferation (Fig 2).

### AT2R activation increased migration and tube formation in pregnant-hUAECs

**Fig 3:**
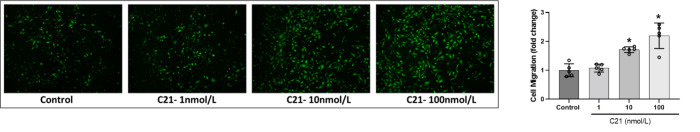
Effect of AT2R agonist C21 on migration of pregnant-hUAECs. Cells were treated with increasing concentrations of C21 (1–100 nmol/L) for 24 h and chemotactic mobility was measured with FluoroBlok Assay. The migrated cells on the bottom of the insert were stained with calcein AM (0.2 μg/ml), and fluorescence images were captured with an inverted microscope (shown at left). The migrated cells were quantified with a fluorescence microplate reader (shown at right). Data were expressed as mean ± SD from five independent experiments using pregnant-hUAECs from five donors and presented as fold of control. *P < 0.05 vs control.

Since C21 induced proliferation only in pregnant-hUAECs, we further focused on these cells to determine if AT2R activation induced cell migration and capillary-like tube formation. Assessment of chemotactic motility using FluoroBlok transwell assay showed that C21 induced cell migration in a concentration-dependent manner with 1, 10, and 100 nmol/L inducing 1.2-, 1.7-, and 2.2-fold increases in cell migration, respectively, in pregnant-hUAECs (Fig 3).

**Fig 4:**
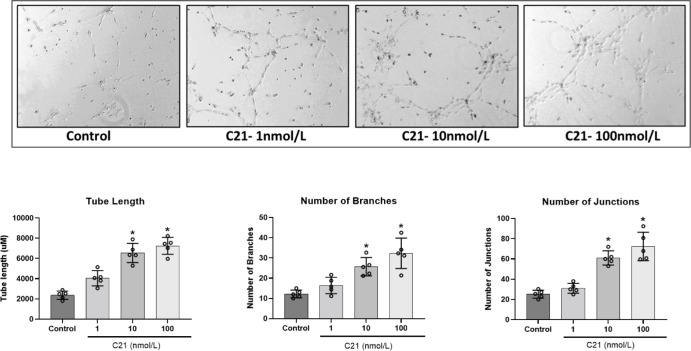
Effect of AT2R agonist C21 on tube formation in pregnant-hUAECs. Cells were seeded in 48-well plates on a semi-solid Matrigel matrix and treated with an increasing concentration of C21 (1–100 nmol/L) for 8 h. Images were captured with a MicroPublisher 5.0 camera and quantification was performed with an ImageJ Angiogenesis Analyzer tool. Representative images are shown at the top and quantified tube length, number of branches, and number of junctions are shown at the bottom. Data were expressed as mean ± SD from five independent experiments using hUAECs from five donors. *P < 0.05 vs control.

Capillary-like tube formation was assessed with Matrigel matrix. As shown in Fig 3, increasing concentrations of C21 (1, 10, 100 nmol/L) resulted in an increased amount of tubular structures in pregnant-hUAECs. Capillary tube length was 2370 ± 119.3 μm in controls, and C21 increased mean tube length to 4050 ± 319.3 μm at 1 nmol/L C21, 6550 ± 476.7 μm at 10 nmol/L C21, and 7245 ± 308.7 μm at 100 nmol/L C21. Similarly, increasing the concentration of C21 increased the total number of branches (control: 13.0 ± 1.9; 1 nmol/L: 16.4 ± 2.1; 10 nmol/L: 25.6 ± 2.7; 100 nmol/L: 32.3 ± 6.8) and total number of junctions (control: 25.2 ± 2.6; 1 nmol/L: 31.0 ± 3.5; 10 nmol/L: 61.0 ± 10.9; 100 nmol/L: 72.3 ± 11.5) in pregnant-hUAECs (Fig 4).

### AT2R activation-increased expression of angiogenic proteins in pregnant-hUAECs

**Fig 5:**
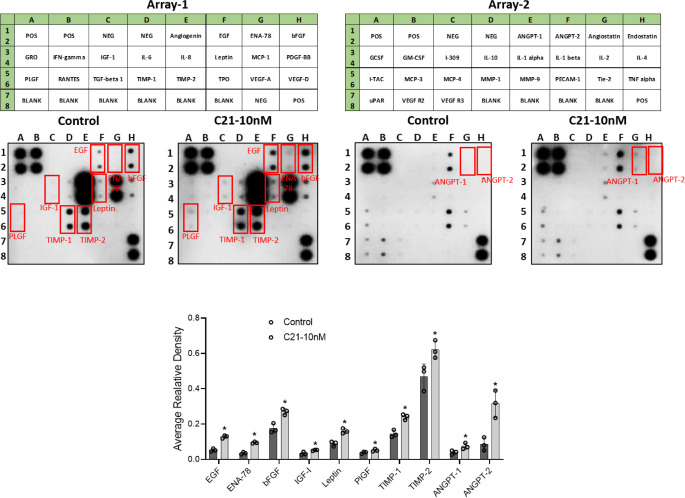
Effect of AT2R agonist C21 on the expression of angiogenic proteins in pregnant-hUAECs. The top panel presents the layout of the Human Antibody Angiogenesis Array (RayBiotech, AAH-ANG-1 and 2), including the positions of each protein. Representative images of human angiogenesis array membranes are shown in the middle panel. Normalized densitometric quantification of the arrays is shown in the bottom panel. Data were expressed as mean ± SD from three independent experiments using hUAECs from three donors. *P < 0.05 vs control.

Lacking any clues about which angiogenic factors might possibly be involved in the above phenomenon, we resolved to screen the differential expression of angiogenesis-related protein targets in pregnant-hUAECs after the 10 nmol/L C21 treatment. We used a membrane-based antibody array to quantify 43 proteins representing growth and differentiation factors, membrane-bound receptors, extracellular matrix components, and intracellular signaling molecules. Of the 43 array proteins tested, 24 were detectable in pregnant-hUAECs. Among these, C21 significantly increased the expression of 10 angiogenic proteins: growth factors (EGF, ENA-78, bFGF, IGF-1, PLGF, angiopoietin-1, angiopoietin-2), TIMPs (TIMP-1, TIMP-2), and adipokines (leptin) (Fig 5). The respective positions of these proteins on the membrane are shown in the top panel, and representative blots are shown in the middle panel (Fig 5).

### AT2R activation induced expression of angiogenic proteins at the transcriptional level

**Fig 6:**
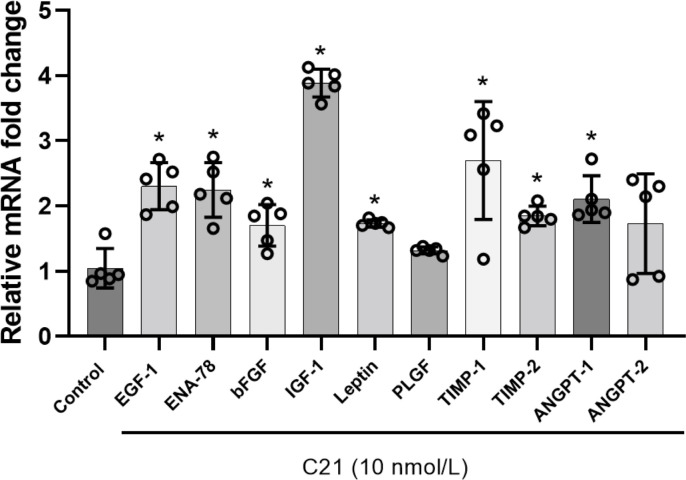
Effect of AT2R agonist C21 on the mRNA expression of angiogenic genes in pregnant-hUAECs. Cells were cultured in an endothelial cell medium containing vehicle or C21 (10 nmol/L) for 24 h, real-time PCR was used to assess gene expression, and data were normalized with GAPDH. Values are expressed as mean ± SD from five independent experiments. *P < 0.05 vs control.

We next used qPCR analysis to examine whether C21-induced increase in expression of angiogenic proteins occurs at the transcriptional level. C21 (10 nmol/L) increased the mRNA expression of EGF (2.3-fold), ENA-78 (2.2-fold), bFGF (1.7-fold), IGF-1 (3.8-fold), leptin (1.7-fold), PLGF (1.3-fold), TIMP-1 (2.7-fold), TIMP-2 (1.8-fold), angiopoietin-1 (2.1-fold), and angiopoietin-2 (2.1-fold) (Fig 6), indicating that AT2R activation upregulates angiogenic proteins at the mRNA level.

## Discussion

The main aim of this study has been to investigate the angiogenic potential of AT2R activation in hUAECs isolated from normal nonpregnant and pregnant women. Our results indicate that AT2R agonist C21 significantly increased proliferation *in vitro* in pregnant-hUAECs, but not nonpregnant-hUAECs. In addition, JNK appears to transduce intracellular signals that regulate AT2R-stimulated mitogenic responses in pregnant-hUAECs. Finally, AT2R activation induced both migration and tube formation in pregnant-hUAECs, with associated increases in the mRNA and protein levels of ten proangiogenic proteins: EGF, ENA-78, bFGF, IGF-1, leptin, PLGF, TIMP-1, TIMP-2, and angiopoietin-1 and -2. These results indicate for the first time that AT2R agonist C21 induces pregnancy-dependent JNK-mediated angiogenesis in hUAECs with an associated transcriptional upregulation of multiple angiogenic proteins.

Endothelial cell proliferation is important for the promotion of angiogenesis. We demonstrated that C21 induced the proliferation of hUAECs isolated from pregnant—but not nonpregnant—women, indicating that pregnancy augments AT2R-mediated responses in hUAECs. The finding that pretreatment with the AT2R antagonist inhibited C21-induced cell proliferation in pregnant-hUAECs confirms that C21 mediates its effects through AT2R as reported previously [23, 24]. The inability of AT2R activation to induce proliferative responses in cells from the nonpregnant state is in line with observations regarding CGP42112A (another AT2R agonist) stimulation of primary bovine retinal endothelial cells and rat coronary endothelial cells [25, 26]. Whether the higher AT2R expression in pregnant-hUAECs relative to nonpregnant-hUAECs [13, 14] contributes to differential mitogenic responses is unclear. However, such a contribution seems unlikely because previous studies has shown that *in vitro* overexpression of AT2R in hepatocellular carcinoma cells induce marked anti-proliferative and apoptotic responses [27]. The observation that high (≥5%) serum induces proliferation in both pregnant- and nonpregnant-hUAECs [18] suggests that nonpregnant-hUAECs have proliferative capacity, but that pregnancy may reprogram the hUAECs in a way that intensifies their response to certain mitogenic agonists and related factors. This suggestion is supported by the observation that estradiol and VEGF induce mitogenic responses only in pregnant-UAECs, but not nonpregnant-UAECs [18, 28]. The mechanistic significance of pregnancy-inducing endothelial-cell adaptation favoring enhanced proliferative responses to certain agonists/factors remains to be elucidated.

Inhibition of ERK1/2, p38, and PI3K failed to reverse the C21-mediated proliferation of pregnant‐hUAECs, thereby ruling out the potential role of these pathways. Another interesting finding herein was the observation that activation of JNK is needed for AT2R‐mediated proliferation of pregnant‐hUAECs, as treatment with SP600125 completely inhibited the proliferation induced by C21. Our results are not consistent with previous reports, since AT2R signaling is known to be mediated by activation of multiple pathways, including p38 MAPK in rat lumbar dorsal root ganglia [29], ERK1/2 in human fibroblasts [30], and PI3K in porcine proximal tubule cells [31]. In addition, AT2R activation inhibited JNK signaling in mice vascular smooth muscle cells [32]. Interestingly, all these studies used cells from the nonpregnant state, in which the expression profiles of kinases and their signaling are very different from those present in the pregnant state [33–35]. Consistent with our finding, other studies have reported that activation of JNK is required for proliferative responses in UAEC isolated from pregnant sheep [21, 22]. Thus, JNK signaling pathways—but not ERK1/2, p38, and PI3K signaling pathways—are involved in pregnant‐hUAEC proliferation mediated by AT2R. Future studies should examine how AT2R couples with and activates JNK signaling in pregnant-hUAECs.

Cell migration is another essential feature of vascular endothelial cells during angiogenesis. To detect migration and angiogenesis ability *in vitro*, we performed transwell migration and tube-formation assay. The results confirm that AT2R activation with C21 can enhance cell migration and the tube-forming ability in pregnant-hUAECs. In trying to better understand the mechanisms of angiogenesis orchestrated by AT2R activation, we examined angiogenic proteins affected by C21 treatment. According to the results of the antibody-array analysis, pregnant-hUAECs treated with C21 exhibited a significant increase in the levels of proangiogenic cytokines and chemokines (EGF, ENA-78, bFGF, IGF-1, PLGF, angiopoietin-1, angiopoietin-2), TIMPs (TIMP-1, TIMP-2), and adipokines (leptin). Previous research has shown that EGF signaling plays a role in the maturation of endothelial tubes and the recruitment of smooth muscle cells [36]. Moreover, other research has shown that bFGF is a positive regulator of angiogenesis [37]. In addition to its mitogenic effect on endothelial cells *in vitro* and *in vivo*, bFGF signaling is critically important for the formation and maintenance of vessels at the uteroplacental interface [38]. Decreased bFGF levels were observed in pregnant women who subsequently developed preeclampsia [39]. PlGF is a member of the VEGF sub-family and is a potent angiogenic factor [40]. In its vital contribution to pregnancy, PIGF specifically promotes the induction of vessel growth and the proliferation, migration, and survival of endothelial cells in various tissues [41, 42]. Reduced levels of PIGF have been reported in preeclampsia [43]. IGF1 increases amino-acid uptake, glucose uptake, and migration and tube formation in endothelial cells [44, 45]. Angiopoietins are critical for vessel homeostasis and angiogenesis. They not only induce vessel maturation, sprouting angiogenesis, and the stability of newly formed vessels, but inhibit pathological vascular permeability, as well [46]. TIMPs are a key regulator of the extracellular matrix that is important in maintaining the connections between endothelial cells [47]. The C21-induced increase in expression of these key angiogenic proteins suggests that AT2R activation may help in the formation of new blood vessels. The qPCR finding that C21 upregulates mRNA levels of these angiogenic proteins suggests that transcriptional mechanisms are involved in the upregulation of the corresponding proangiogenic proteins. Further studies are needed to determine how AT2R induces transcription of the angiogenic genes. In the present study, using angiogenic transcriptomic and proteomic analyses, we have shown that C21 commonly regulates a family of genes associated with angiogenesis, suggesting that AT2R plays a cooperative role in orchestrating an important proangiogenic transcriptional program in pregnant-hUAECs.

In conclusion, in the present study, we have demonstrated that AT2R activation induces proliferation of hUAECs in a pregnancy-specific manner through JNK-mediated signaling with associated transcriptional upregulation of multiple proangiogenic proteins. Vascular AT2R expression is decreased in pregnancy disorders like preeclampsia and fetal growth restriction [48, 49]. The findings of this study showing that AT2R promotes angiogenesis, together with previous reports that AT2R stimulation upregulates expression of its own receptors and enhances uterine blood flow [14], suggest that the AT2R agonist could be a new class of therapeutic agents capable of curtailing pregnancy disorders that involve impaired angiogenesis.

## References

[1] Risau W. Mechanisms of angiogenesis. Nature. 1997;386(6626):671–4. Epub 1997/04/17. doi: 10.1038/386671a0 . 9109485

[2] Magness RR. Maternal cardiovascular and other physiologic responses to the endocrinology of pregnancy. In Bazer FW, ed The Endocrinology of Pregnancy Totowa, NJ: Humana Press Inc. 1998:507–39.

[3] Rosenfeld CR. Distribution of cardiac output in ovine pregnancy. Am J Physiol. 1977;232(3):H231–5. Epub 1977/03/01. doi: 10.1152/ajpheart.1977.232.3.H231 . 842676

[4] Barut F, Barut A, Gun BD, Kandemir NO, Harma MI, Harma M, et al. Intrauterine growth restriction and placental angiogenesis. Diagn Pathol. 2010;5:24. Epub 2010/04/24. doi: 10.1186/1746-1596-5-24 ; PubMed Central PMCID: PMC2865442. 20412591 PMC2865442

[5] Myatt L. Role of placenta in preeclampsia. Endocrine. 2002;19(1):103–11. Epub 2003/02/14. doi: 10.1385/ENDO:19:1:103 . 12583607

[6] Ahmed A, Dunk C, Ahmad S, Khaliq A. Regulation of placental vascular endothelial growth factor (VEGF) and placenta growth factor (PIGF) and soluble Flt-1 by oxygen—a review. Placenta. 2000;21 Suppl A:S16–24. Epub 2000/06/01. doi: 10.1053/plac.1999.0524 . 10831117

[7] Lu Y, Sun X, Peng L, Jiang W, Li W, Yuan H, et al. Angiotensin II-Induced vascular remodeling and hypertension involves cathepsin L/V- MEK/ERK mediated mechanism. Int J Cardiol. 2020;298:98–106. Epub 2019/11/02. doi: 10.1016/j.ijcard.2019.09.070 . 31668507

[8] Shah DM. Preeclampsia: new insights. Curr Opin Nephrol Hypertens. 2007;16(3):213–20. Epub 2007/04/11. doi: 10.1097/MNH.0b013e3280d942e9 . 17420664

[9] Munk VC, Sanchez de Miguel L, Petrimpol M, Butz N, Banfi A, Eriksson U, et al. Angiotensin II induces angiogenesis in the hypoxic adult mouse heart in vitro through an AT2-B2 receptor pathway. Hypertension. 2007;49(5):1178–85. Epub 2007/03/07. doi: 10.1161/HYPERTENSIONAHA.106.080242 . 17339539

[10] Mishra JS, Kumar S. Activation of angiotensin type 2 receptor attenuates testosterone-induced hypertension and uterine vascular resistance in pregnant ratsdagger. Biol Reprod. 2021;105(1):192–203. Epub 2021/03/20. doi: 10.1093/biolre/ioab051 ; PubMed Central PMCID: PMC8660162. 33739377 PMC8660162

[11] Williams PJ, Mistry HD, Innes BA, Bulmer JN, Broughton Pipkin F. Expression of AT1R, AT2R and AT4R and their roles in extravillous trophoblast invasion in the human. Placenta. 2010;31(5):448–55. Epub 2010/03/23. doi: 10.1016/j.placenta.2010.02.014 . 20304486

[12] Burrell JH, Lumbers ER. Angiotensin receptor subtypes in the uterine artery during ovine pregnancy. Eur J Pharmacol. 1997;330(2–3):257–67. Epub 1997/07/09. doi: 10.1016/s0014-2999(97)00167-2 . 9253961

[13] Mishra JS, Te Riele GM, Qi QR, Lechuga TJ, Gopalakrishnan K, Chen DB, et al. Estrogen Receptor-beta Mediates Estradiol-Induced Pregnancy-Specific Uterine Artery Endothelial Cell Angiotensin Type-2 Receptor Expression. Hypertension. 2019;74(4):967–74. Epub 2019/08/06. doi: 10.1161/HYPERTENSIONAHA.119.13429 ; PubMed Central PMCID: PMC6739159. 31378106 PMC6739159

[14] Mishra JS, Gopalakrishnan K, Kumar S. Pregnancy upregulates angiotensin type 2 receptor expression and increases blood flow in uterine arteries of rats. Biol Reprod. 2018;99(5):1091–9. Epub 2018/06/04. doi: 10.1093/biolre/ioy130 ; PubMed Central PMCID: PMC6297314. 29860295 PMC6297314

[15] Mirabito KM, Hilliard LM, Wei Z, Tikellis C, Widdop RE, Vinh A, et al. Role of inflammation and the angiotensin type 2 receptor in the regulation of arterial pressure during pregnancy in mice. Hypertension. 2014;64(3):626–31. Epub 2014/06/18. doi: 10.1161/HYPERTENSIONAHA.114.03189 . 24935937

[16] St-Louis J, Sicotte B, Bedard S, Brochu M. Blockade of angiotensin receptor subtypes in arcuate uterine artery of pregnant and postpartum rats. Hypertension. 2001;38(5):1017–23. Epub 2001/11/17. doi: 10.1161/hy1101.095008 . 11711491

[17] Wan Y, Wallinder C, Plouffe B, Beaudry H, Mahalingam AK, Wu X, et al. Design, synthesis, and biological evaluation of the first selective nonpeptide AT2 receptor agonist. J Med Chem. 2004;47(24):5995–6008. Epub 2004/11/13. doi: 10.1021/jm049715t . 15537354

[18] Zhang HH, Chen JC, Sheibani L, Lechuga TJ, Chen DB. Pregnancy Augments VEGF-Stimulated In Vitro Angiogenesis and Vasodilator (NO and H2S) Production in Human Uterine Artery Endothelial Cells. J Clin Endocrinol Metab. 2017;102(7):2382–93. Epub 2017/04/12. doi: 10.1210/jc.2017-00437 ; PubMed Central PMCID: PMC5505189. 28398541 PMC5505189

[19] Liao WX, Feng L, Zhang H, Zheng J, Moore TR, Chen DB. Compartmentalizing VEGF-induced ERK2/1 signaling in placental artery endothelial cell caveolae: a paradoxical role of caveolin-1 in placental angiogenesis in vitro. Mol Endocrinol. 2009;23(9):1428–44. Epub 2009/05/30. doi: 10.1210/me.2008-0475 ; PubMed Central PMCID: PMC2737550. 19477952 PMC2737550

[20] Zhang D, Tang HY, Tan L, Zhao DM. MALAT1 is involved in the pathophysiological process of PCOS by modulating TGFbeta signaling in granulosa cells. Mol Cell Endocrinol. 2020;499:110589. Epub 2019/09/27. doi: 10.1016/j.mce.2019.110589 . 31557499

[21] Hu XQ, Zhang L. Angiogenesis during pregnancy: all routes lead to MAPKs. J Physiol. 2017;595(14):4571–2. Epub 2017/05/18. doi: 10.1113/JP274489 ; PubMed Central PMCID: PMC5509881. 28513857 PMC5509881

[22] Landeros RV, Jobe SO, Aranda-Pino G, Lopez GE, Zheng J, Magness RR. Convergent ERK1/2, p38 and JNK mitogen activated protein kinases (MAPKs) signalling mediate catecholoestradiol-induced proliferation of ovine uterine artery endothelial cells. J Physiol. 2017;595(14):4663–76. Epub 2017/04/25. doi: 10.1113/JP274119 ; PubMed Central PMCID: PMC5509880. 28437005 PMC5509880

[23] Sampson AK, Irvine JC, Shihata WA, Dragoljevic D, Lumsden N, Huet O, et al. Compound 21, a selective agonist of angiotensin AT2 receptors, prevents endothelial inflammation and leukocyte adhesion in vitro and in vivo. Br J Pharmacol. 2016;173(4):729–40. Epub 2015/01/07. doi: 10.1111/bph.13063 ; PubMed Central PMCID: PMC4742292. 25560767 PMC4742292

[24] Foulquier S, Steckelings UM, Unger T. Impact of the AT(2) receptor agonist C21 on blood pressure and beyond. Curr Hypertens Rep. 2012;14(5):403–9. Epub 2012/07/28. doi: 10.1007/s11906-012-0291-6 . 22836386

[25] Carbajo-Lozoya J, Lutz S, Feng Y, Kroll J, Hammes HP, Wieland T. Angiotensin II modulates VEGF-driven angiogenesis by opposing effects of type 1 and type 2 receptor stimulation in the microvascular endothelium. Cell Signal. 2012;24(6):1261–9. Epub 2012/03/01. doi: 10.1016/j.cellsig.2012.02.005 . 22374305

[26] Stoll M, Steckelings UM, Paul M, Bottari SP, Metzger R, Unger T. The angiotensin AT2-receptor mediates inhibition of cell proliferation in coronary endothelial cells. J Clin Invest. 1995;95(2):651–7. Epub 1995/02/01. doi: 10.1172/JCI117710 ; PubMed Central PMCID: PMC295531. 7860748 PMC295531

[27] Du H, Liang Z, Zhang Y, Jie F, Li J, Fei Y, et al. Effects of angiotensin II type 2 receptor overexpression on the growth of hepatocellular carcinoma cells in vitro and in vivo. PLoS One. 2013;8(12):e83754. Epub 2014/01/07. doi: 10.1371/journal.pone.0083754 ; PubMed Central PMCID: PMC3877089. 24391821 PMC3877089

[28] Jobe SO, Ramadoss J, Koch JM, Jiang Y, Zheng J, Magness RR. Estradiol-17beta and its cytochrome P450- and catechol-O-methyltransferase-derived metabolites stimulate proliferation in uterine artery endothelial cells: role of estrogen receptor-alpha versus estrogen receptor-beta. Hypertension. 2010;55(4):1005–11. Epub 2010/03/10. doi: 10.1161/HYPERTENSIONAHA.109.146399 ; PubMed Central PMCID: PMC2876348. 20212268 PMC2876348

[29] Smith MT, Woodruff TM, Wyse BD, Muralidharan A, Walther T. A small molecule angiotensin II type 2 receptor (AT(2)R) antagonist produces analgesia in a rat model of neuropathic pain by inhibition of p38 mitogen-activated protein kinase (MAPK) and p44/p42 MAPK activation in the dorsal root ganglia. Pain Med. 2013;14(10):1557–68. Epub 2013/06/08. doi: 10.1111/pme.12157 . 23742186

[30] Calo LA, Schiavo S, Davis PA, Pagnin E, Mormino P, D’Angelo A, et al. Angiotensin II signaling via type 2 receptors in a human model of vascular hyporeactivity: implications for hypertension. J Hypertens. 2010;28(1):111–8. Epub 2009/10/03. doi: 10.1097/HJH.0b013e328332b738 . 19797979

[31] Caruso-Neves C, Kwon SH, Guggino WB. Albumin endocytosis in proximal tubule cells is modulated by angiotensin II through an AT2 receptor-mediated protein kinase B activation. Proc Natl Acad Sci U S A. 2005;102(48):17513–8. Epub 2005/11/19. doi: 10.1073/pnas.0507255102 ; PubMed Central PMCID: PMC1297674. 16293694 PMC1297674

[32] Matsubara H, Shibasaki Y, Okigaki M, Mori Y, Masaki H, Kosaki A, et al. Effect of angiotensin II type 2 receptor on tyrosine kinase Pyk2 and c-Jun NH2-terminal kinase via SHP-1 tyrosine phosphatase activity: evidence from vascular-targeted transgenic mice of AT2 receptor. Biochem Biophys Res Commun. 2001;282(5):1085–91. Epub 2001/04/17. doi: 10.1006/bbrc.2001.4695 . 11302725

[33] Grummer MA, Sullivan JA, Magness RR, Bird IM. Vascular endothelial growth factor acts through novel, pregnancy-enhanced receptor signalling pathways to stimulate endothelial nitric oxide synthase activity in uterine artery endothelial cells. Biochem J. 2009;417(2):501–11. Epub 2008/09/26. doi: 10.1042/BJ20081013 ; PubMed Central PMCID: PMC2680709. 18816248 PMC2680709

[34] Sullivan JA, Grummer MA, Yi FX, Bird IM. Pregnancy-enhanced endothelial nitric oxide synthase (eNOS) activation in uterine artery endothelial cells shows altered sensitivity to Ca2+, U0126, and wortmannin but not LY294002—evidence that pregnancy adaptation of eNOS activation occurs at multiple levels of cell signaling. Endocrinology. 2006;147(5):2442–57. Epub 2006/02/04. doi: 10.1210/en.2005-0399 . 16455784

[35] Bird IM, Sullivan JA, Di T, Cale JM, Zhang L, Zheng J, et al. Pregnancy-dependent changes in cell signaling underlie changes in differential control of vasodilator production in uterine artery endothelial cells. Endocrinology. 2000;141(3):1107–17. Epub 2000/03/04. doi: 10.1210/endo.141.3.7367 . 10698187

[36] van Cruijsen H, Giaccone G, Hoekman K. Epidermal growth factor receptor and angiogenesis: Opportunities for combined anticancer strategies. Int J Cancer. 2005;117(6):883–8. Epub 2005/09/10. doi: 10.1002/ijc.21479 . 16152621

[37] Nakamichi M, Akishima-Fukasawa Y, Fujisawa C, Mikami T, Onishi K, Akasaka Y. Basic Fibroblast Growth Factor Induces Angiogenic Properties of Fibrocytes to Stimulate Vascular Formation during Wound Healing. Am J Pathol. 2016;186(12):3203–16. Epub 2016/10/25. doi: 10.1016/j.ajpath.2016.08.015 . 27773739

[38] Martinez-Fierro ML, Hernadez-Delgadillo GP, Flores-Mendoza JF, Alvarez-Zuniga CD, Diaz-Lozano ML, Delgado-Enciso I, et al. Fibroblast Growth Factor Type 2 (FGF2) Administration Attenuated the Clinical Manifestations of Preeclampsia in a Murine Model Induced by L-NAME. Front Pharmacol. 2021;12:663044. Epub 2021/05/08. doi: 10.3389/fphar.2021.663044 ; PubMed Central PMCID: PMC8093788. 33959027 PMC8093788

[39] Martinez-Fierro ML, Castruita-De La Rosa C, Garza-Veloz I, Cardiel-Hernandez RM, Espinoza-Juarez MA, Delgado-Enciso I, et al. Early pregnancy protein multiplex screening reflects circulating and urinary divergences associated with the development of preeclampsia. Hypertens Pregnancy. 2018;37(1):37–50. Epub 2018/01/09. doi: 10.1080/10641955.2017.1411946 . 29308696

[40] Athanassiades A, Lala PK. Role of placenta growth factor (PIGF) in human extravillous trophoblast proliferation, migration and invasiveness. Placenta. 1998;19(7):465–73. Epub 1998/10/20. doi: 10.1016/s0143-4004(98)91039-6 . 9778119

[41] Carmeliet P, Moons L, Luttun A, Vincenti V, Compernolle V, De Mol M, et al. Synergism between vascular endothelial growth factor and placental growth factor contributes to angiogenesis and plasma extravasation in pathological conditions. Nat Med. 2001;7(5):575–83. Epub 2001/05/01. doi: 10.1038/87904 . 11329059

[42] Ziche M, Maglione D, Ribatti D, Morbidelli L, Lago CT, Battisti M, et al. Placenta growth factor-1 is chemotactic, mitogenic, and angiogenic. Lab Invest. 1997;76(4):517–31. Epub 1997/04/01. . 9111514

[43] Chau K, Hennessy A, Makris A. Placental growth factor and pre-eclampsia. J Hum Hypertens. 2017;31(12):782–6. Epub 2017/11/09. doi: 10.1038/jhh.2017.61 ; PubMed Central PMCID: PMC5680413. 29115294 PMC5680413

[44] Bach LA. Endothelial cells and the IGF system. J Mol Endocrinol. 2015;54(1):R1–13. Epub 2014/10/30. doi: 10.1530/JME-14-0215 . 25351818

[45] Grant M, Jerdan J, Merimee TJ. Insulin-like growth factor-I modulates endothelial cell chemotaxis. J Clin Endocrinol Metab. 1987;65(2):370–1. Epub 1987/08/01. doi: 10.1210/jcem-65-2-370 . 3597713

[46] Fagiani E, Christofori G. Angiopoietins in angiogenesis. Cancer Lett. 2013;328(1):18–26. Epub 2012/08/28. doi: 10.1016/j.canlet.2012.08.018 . 22922303

[47] Rojiani MV, Ghoshal-Gupta S, Kutiyanawalla A, Mathur S, Rojiani AM. TIMP-1 overexpression in lung carcinoma enhances tumor kinetics and angiogenesis in brain metastasis. J Neuropathol Exp Neurol. 2015;74(4):293–304. Epub 2015/03/11. doi: 10.1097/NEN.0000000000000175 . 25756591

[48] Yamaleyeva LM, Neves LA, Coveleskie K, Diz DI, Gallagher PE, Brosnihan KB. AT1, AT2, and AT(1–7) receptor expression in the uteroplacental unit of normotensive and hypertensive rats during early and late pregnancy. Placenta. 2013;34(6):497–502. Epub 2013/04/23. doi: 10.1016/j.placenta.2013.03.008 ; PubMed Central PMCID: PMC3647340. 23602334 PMC3647340

[49] Judson JP, Nadarajah VD, Bong YC, Subramaniam K, Sivalingam N. A preliminary finding: immunohistochemical localisation and distribution of placental angiotensin II receptor subtypes in normal and preeclamptic pregnancies. Med J Malaysia. 2006;61(2):173–80. Epub 2006/08/11. . 16898308

